# Advanced Airway Device Use Order During Out-of-Hospital Cardiac Arrest

**DOI:** 10.1001/jamanetworkopen.2025.53413

**Published:** 2026-01-12

**Authors:** Christopher B. Gage, Jacob C. Kamholz, Jonathan R. Powell, Michelle M. J. Nassal, Henry E. Wang, Ashish R. Panchal

**Affiliations:** 1 Division of Epidemiology, The Ohio State University College of Public Health, Columbus; 2 Division of Health Services Management & Policy, The Ohio State University College of Public Health, Columbus; 3 Research Team, National Registry of Emergency Medical Technicians, Columbus, Ohio; 4 Research, ImageTrend Inc, Eagan, Minnesota; 5 Department of Emergency Medicine, The Ohio State University, Columbus

## Abstract

**Importance:**

During out-of-hospital cardiac arrest (OHCA), initial airway device choice as well as final device choice in the event of failure may both influence overall procedural success and patient outcomes. Understanding national practice patterns in airway device placement may inform resuscitation strategies and system-level quality improvement.

**Objective:**

To assess patterns in the order of advanced airway device use for adults in the United States receiving OHCA treatment during emergency medical service (EMS) activations.

**Design, Setting, and Participants:**

This retrospective cross-sectional study used data from the National EMS Information System (NEMSIS) database from January 2018 to December 2023. During this period, NEMSIS included more than 256 million EMS activations from nearly 14 000 agencies across 54 states and territories. Activations for 911 responses during adult OHCA with advanced airway device attempts after EMS arrival, with documented success (yes or no) and number of attempts, were included.

**Exposures:**

Advanced airway devices documented by EMS clinicians.

**Main Outcome and Measures:**

Order of advanced airway device use, measured by EMS clinician documentation.

**Results:**

From 2018 to 2023, EMS reported 2 365 224 activations involving adults having an OHCA, with 650 440 patients (0.3%) receiving either endotracheal intubation (ETI) and/or supraglottic airway (SGA). Most patients received a successful initial airway device attempt (503 774 [77.4%]). Although ETI was the most common initial airway device (458 546 [70.5%]), SGA had a higher first-pass success rate (93.0% vs 71.0%). Among patients whose first ETI attempt failed (133 177 of 458 546 [29.0%]), the final documented device remained ETI in 72 167 cases (72.3%) and changed to SGA in 27 651 (27.7%). Among patients whose first SGA attempt failed, 13 480 (61.9%) remained with SGA and 3388 (38.1%) were switched to ETI.

**Conclusions and Relevance:**

In this cross-sectional study, most patients with OHCA received ETI as the first advanced airway procedure, despite higher SGA first-pass success. Among patients whose initial attempt failed, most continued with the same device. However, switching from ETI to SGA had a higher final success rates. These findings highlight the importance of airway device sequence in prehospital care and may inform training and protocols for airway management.

## Key Points

**Question:**

What are national practice patterns in the order of advanced airway device use during adult out-of-hospital cardiac arrest (OHCA) among emergency medical service (EMS) activations?

**Findings:**

In this cross-sectional study of 2 365 224 US EMS activations from 2018 to 2023, adult patients more commonly received endotracheal intubation (ETI) than supraglottic airway (SGA) as the initial advanced airway device. Most patients underwent single device placement, with SGA having higher initial and final device success rates.

**Meaning:**

In this study, most patients with OHCA received a successful airway with only a single advanced airway intervention, with SGAs having higher initial and final device success rates than ETI.

## Teaser

This cross-sectional study examines national patterns in the order in which emergency medical services clinicians use advanced airway support devices, namely endotracheal intubation and supraglottic airway, among US adults with out-of-hospital cardiac arrest.

## Introduction

Endotracheal intubation (ETI) is the most common advanced airway management choice in adult out-of-hospital cardiac arrest (OHCA).^1,2,3,4,5^ With the ability to secure a definitive airway and facilitate effective ventilation, ETI remains a cornerstone of high-acuity resuscitation. However, despite historical precedence, ETI is technically challenging and requires substantial training and experience to perform successfully in prehospital settings, contributing to variations in success rates and outcomes across different emergency medical service (EMS) systems.^6,7^ Additionally, ETI is inherently difficult during cardiac arrest with the potential to interrupt chest compressions and other critical interventions.^8,9^

Recently, supraglottic airway (SGA) device use has increased as an alternative to ETI for patients with OHCA.^1,10^ SGAs are often easier and faster to insert, with fewer technical demands, allowing a broader range of EMS clinicians to perform advanced airway management successfully.^11,12^ As SGA use becomes more widespread, questions have emerged about how availability of multiple airway devices may influence prehospital airway management practices.^6^

Most studies have focused on comparing airway devices by type, assessing either the final airway placed or the overall success, without accounting for the order in which devices were used.^5^ However, the sequence in which devices are used, including whether clinicians persist with the initial device or subsequent switches after a failed attempt, may influence overall airway success and reflect different clinical decision-making strategies during resuscitation. The objective of this study was to evaluate the order of airway device use (ie, initial device and final device choice in the event of failure) in adult patients with OHCA and examine whether success rates differ by device type and sequence.

## Methods

### Study Design

This retrospective cross-sectional study assessed adult patients with OHCA receiving an advanced airway between 2018 and 2023. The American Institutes for Research Institutional Review Board deemed the study exempt from review and the requirement for informed consent due to the use of deidentified data. We followed the Strengthening the Reporting of Observational Studies in Epidemiology (STROBE) reporting guideline for cross-sectional studies.

### Setting

Data were obtained from the National Emergency Medical Services Information System (NEMSIS), a national repository of standardized EMS patient care records in the United States.^13^ NEMSIS was established in 2001 through a collaboration between the National Association of State EMS Directors, the National Highway Traffic Safety Administration, and the Health Resources and Services Administration.^13^ It aims to improve the quality and efficiency of EMS care by enabling consistent data collection, aggregation, and public use for research and policy development. NEMSIS comprises 585 data elements, 165 of which are mandatory at the national level, covering clinical care metrics (eg, dispatch and scene times) and activities (eg, procedures and medications) from dispatch to disposition.^14^ The dataset represents EMS operations for approximately 95% of EMS agencies that handle 911 calls for emergency care and transport to acute care facilities. The NEMSIS Technical Assistance Center receives data from 75% of all electronic patient care reports (ePCRs) generated daily in the United States, with more than 99% of ePCRs submitted within 10 days of patient contact.^15^ From January 2018 to December 2023, NEMSIS included more than 256 million EMS activations submitted by nearly 14 000 agencies across 54 US states and territories.^15^ NEMSIS Data Dictionary version 3.4 was used to identify codes for the variables used in this analysis.^16^

### Population

Similar to previous evaluations, we identified adult patients (age ≥18 years) from activations initiated through 911 responses who received an advanced airway device after EMS arrival.^1,3,17^ We included patients if clinicians documented both the number of airway attempts (NEMSIS variable eProcedures.05) and procedure success (NEMSIS variable eProcedures.06). We classified cases as OHCA if activations met at least 1 of the following: (1) EMS clinicians performed cardiopulmonary resuscitation (CPR), (2) CPR was documented as occurring before or after EMS arrival, (3) chest compressions were recorded as a procedure, or (4) defibrillation or cardioversion was performed.^1,17^

### Main Outcome

The main outcome of this study was to evaluate national patterns in the sequence of advanced airway device use, specifically the first and final documented devices (ETI or SGA), among adult patients with OHCA during EMS activations following 911 responses. The secondary outcome was to assess first-pass success by initial airway device and, among patients whose first attempt failed, to determine the success rate of the final documented airway device for each device sequence.

### Measures

Demographic characteristics included patient age (in years; continuous) and sex (male or female). Incident locations are recorded with *International Statistical Classification of Diseases and Related Problems, Tenth Revision* (*ICD-10*) (NEMSIS variable eScene.09*)*, categorized as home or residence, health care facility, non–health care business, street or highway, and other (eg, sporting event, outdoors).

NEMSIS classifies population setting (NEMSIS variable Urbanicity) using the US Department of Agriculture and Office of Management and Budget definitions using Urban Influence Codes (UIC): urban (UIC 1 or 2), counties with large (≥1 million residents) or small (<1 million residents) metropolitan areas; suburban (UIC 3 and 5), micropolitan (with an urban core of ≥10 000 residents) counties adjacent to a large or small metropolitan county; rural (UIC 4, 6, 8, and 9), nonurban core counties neighboring a large metropolitan area or a small metropolitan area (with or without a town); and wilderness (UIC 7, 10, 11, and 12), noncore counties that are adjacent to micropolitan counties (with or without own town).^1,15,18^

Advanced airway devices, either ETI (eg, direct, indirect, video, manual) or SGA (eg, King-LT, i-gel, Combitube), were identified using Systematized Nomenclature of Medicine–Clinical Terms (SNOMED-CT) codes (NEMSIS variable eProcedures.03*)*. The SNOMED-CT codes are standardized, hierarchically organized clinical terminology codes used to consistently document, share, and analyze medical information across health care systems and electronic health records.^19^

Device order was determined using the first and last documented devices based on procedure timestamps (NEMSIS variable eProcedures.01). For patients with multiple documented procedure attempts (NEMSIS variable eProcedures.05) and only 1 outcome recorded as yes (ie, successful; NEMSIS variable eProcedures.06; code, 9923003), we reconstructed the data to reflect each individual attempt as a separate row, assigning the final documented attempt as successful and all preceding attempts as unsuccessful (NEMSIS variable eProcedures.06; code, 9923001). This allowed us to identify first-pass success as the first documented attempt marked successful and to track whether clinicians persisted with the same device or switched after failure. For example, if a patient underwent 3 ETI attempts with only the third marked successful, all 3 were represented, with the first 2 coded as unsuccessful and the third as successful. This approach enabled identification of the first attempted device, any subsequent switching, and the final successful (or unsuccessful) airway device documented per patient.

### Statistical Analysis

We used descriptive statistics, including frequency, median, and IQR, to evaluate airway device order and success by age, sex, urbanicity, and incident location. Race was not reported due to 18.8% missingness. These analyses were conducted with Stata version 18/MP (StataCorp).

## Results

**Figure 1.**
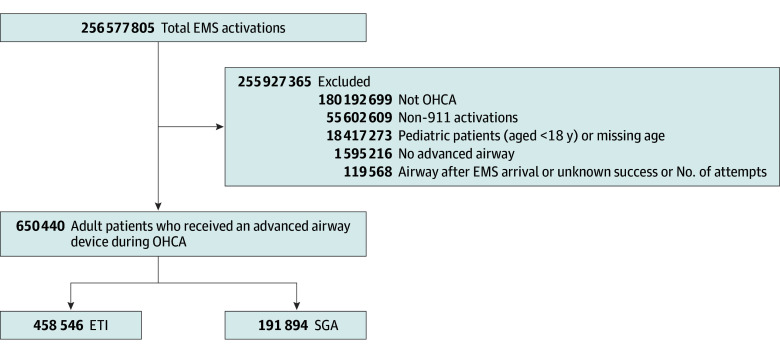
Flow Diagram for Patient Inclusion and Exclusion Criteria From Complete 2018 to 2023 National Emergency Medical Services (EMS) Information System Datasets ETI, endotracheal intubation; OHCA, out-of-hospital cardiac arrest; SGA, supraglottic airway.

A total of 256 577 805 EMS activations took place from 2018 to 2023 in the United States, with 650 440 (0.3%) adult patients who received an advanced airway device during OHCA and included in the analysis (Figure 1). Median (IQR) patient age was 65 (53-76) years, and 410 141 patients (63.1%) were male. Most incidents occurred at home or in a residence (371 611 [57.1%]) and in urban settings (541 816 [83.3%]) (Table).

**Table.** Characteristics of Populations With Out-of-Hospital Cardiac Arrest Receiving an Advanced Airway Device in the National Emergency Medical Services Information System Included in the Case Selection

Characteristic	Patients by year, No. (%)						
	2018 (n = 48 841)	2019 (n = 80 627)	2020 (n = 118 972)	2021 (n = 131 404)	2022 (n = 137 952)	2023 (n = 132 644)	Total (N = 650 440)
Age, median (IQR), y	65 (53-76)	66 (54-76)	65 (53-76)	65 (52-76)	66 (53-76)	66 (53-76)	65 (53-76)
Sex							
Female	17 891 (36.6)	29 666 (36.8)	43 992 (37.0)	48 089 (36.6)	50 663 (36.7)	48 032 (36.2)	238 333 (36.6)
Male	30 733 (62.9)	50 667 (62.8)	74 578 (62.7)	82 894 (63.1)	86 970 (63.0)	84 299 (63.6)	410 141 (63.1)
Missing data	217 (0.4)	294 (0.4)	402 (0.3)	421 (0.3)	319 (0.2)	313 (0.2)	1966 (0.3)
Incident location							
Home or residence	28 718 (58.8)	47 763 (59.2)	68 012 (57.2)	75 389 (57.4)	78 368 (56.8)	73 361 (55.3)	371 611 (57.1)
Health care facility	5471 (11.2)	9398 (11.7)	13 415 (11.3)	14 216 (10.8)	16 024 (11.6)	15 973 (12.0)	74 497 (11.5)
Non–health care business	4267 (8.7)	6618 (8.2)	8544 (7.2)	10 269 (7.8)	10 971 (8.0)	10 813 (8.2)	51 482 (7.9)
Street or highway	3251 (6.7)	5393 (6.7)	7232 (6.1)	8428 (6.4)	8695 (6.3)	8771 (6.6)	41 770 (6.4)
Other (eg, sporting event, outdoors)	1406 (2.9)	2564 (3.2)	2827 (2.4)	3353 (2.6)	3691 (2.7)	3851 (2.9)	17 692 (2.7)
Missing data	5728 (11.7)	8891 (11.0)	18 942 (15.9)	19 749 (15.0)	20 203 (14.6)	19 875 (15.0)	93 388 (14.4)
Urbanicity							
Urban	39 823 (81.5)	66 571 (82.6)	99 638 (83.7)	109 038 (83.0)	115 283 (83.6)	111 463 (84.0)	541 816 (83.3)
Rural	3736 (7.6)	5812 (7.2)	7695 (6.5)	9061 (6.9)	9608 (7.0)	9054 (6.8)	44 966 (6.9)
Suburban	3121 (6.4)	4876 (6.0)	7333 (6.2)	8590 (6.5)	8918 (6.5)	8208 (6.2)	41 046 (6.3)
Wilderness	748 (1.5)	1257 (1.6)	1615 (1.4)	1797 (1.4)	1818 (1.3)	1778 (1.3)	9013 (1.4)
Missing data	1413 (2.9)	2111 (2.6)	2691 (2.3)	2918 (2.2)	2325 (1.7)	2141 (1.6)	13 599 (2.1)

**Figure 2.**
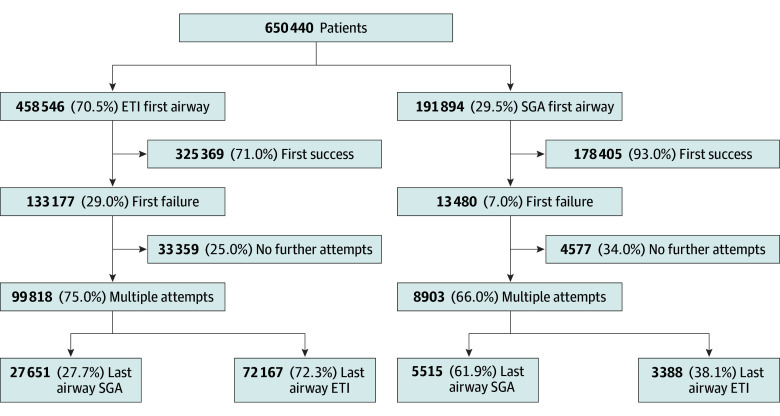
Advanced Airway Device Order Among Adult Patients With Out-of-Hospital Cardiac Arrest Treated With Endotracheal Intubation (ETI) or Supraglottic Airway (SGA) Device, in the National Emergency Medical Services Information System From 2018 to 2023

Among the 650 440 patients, 458 546 (70.5%) received ETI and 191 894 (29.5%) received an SGA as the first documented airway device (Figure 2). First-attempt success was achieved in 325 369 ETI cases (71.0%) and 178 405 SGA cases (93.0%).

**Figure 3.**
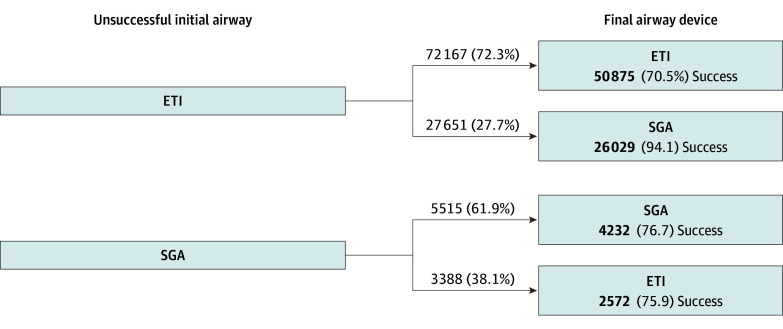
Order and Success of Final Airway Device Following an Unsuccessful Initial Attempt in Adult Out-of-Hospital Cardiac Arrest Cases Reported in the 2018 to 2023 National Emergency Medical Services Information System Datasets ETI indicates endotracheal intubation; SGA, supraglottic airway.

Among patients with an unsuccessful first attempt, 99 818 of 133 177 (75.0%) in the ETI group and 8903 of 13 489 (66.0%) in the SGA group received a subsequent airway device attempt (Figure 3). The remaining patients had no further documented airway attempts following the initial failure: 33 359 (25.0%) in the ETI group and 4577 (33.9%) in the SGA group.

Among the 108 721 patients with multiple airway attempts, 77 682 (71.5%) remained with their initial device type. After a failed first attempt, 27 651 patients with an initial ETI attempt (27.7%) switched to an SGA, while 3388 patients with an initial SGA attempt (38.1%) ultimately switched to ETI.

Among the 99 818 ETI-first patients with additional attempts, 27 651 (27.7%) had a final documented airway of SGA, with a final success rate of 94.1% (26 029 of 27 651). In comparison, 72 167 (72.3%) had a final airway of ETI, with a lower final success rate of 70.5% (50 875 of 72 167). Among the 8903 SGA-first patients with additional attempts, 3388 (38.1%) had a final airway of ETI (final success rate, 75.9%; 2572 of 3388), and 5515 (61.9%) had a final airway of SGA (final success rate, 76.7%; 4232 of 5515).

## Discussion

In this cross-sectional study of more than 650 000 EMS-treated patients with cardiac arrest in the United States from 2018 to 2023, procedural success varied markedly based on the order of advanced airway device use. Among patients whose first ETI attempt failed, those who switched to an SGA had a 94.1% final success rate, higher than the success rate among those who continued with ETI (70.5%). Additionally, most clinicians who failed their initial device placement did not switch to another device: 27 651 ETI-first failures (27.7%) transitioned to SGA, and 3388 SGA-first failures (38.1%) switched to ETI. These findings suggest that EMS clinicians often persist with the initial airway strategy after failure, and that airway device sequence, not just the type, may play a critical role in achieving successful airway management during OHCA.

EMS airway research has largely focused on comparing devices, particularly with respect to outcomes such as first-pass success and survival.^11,20,21^ However, few studies have examined the trajectory of airway management, including what occurs after the first device fails and how subsequent device choice affects success. Two notable examples include a recent Dutch study and the UK-based REVIVE-Airways feasibility trial.^22,23^ The Dutch study^22^ demonstrated that SGA use increased over time, with consistently high first-pass success, but did not detail specific sequences or switching patterns following device failure. In contrast, the REVIVE-Airways trial^23^ showed that two-thirds of patients received multiple airway interventions and that switching devices was common. Our findings extend this conversation by highlighting that device order plays a critical role in clinical judgment: success is not only a function of the initial device chosen but also the persistence in a failed strategy. The observed high initial success of SGA and when switching from ETI to SGA also aligns with trial data, underscoring the SGA’s ease of use and resilience to procedural failure.^11,24^

The decision to use either initial airway device may reflect important clinical considerations, protocols, or clinician preference. While clinically relevant, they are not captured in the current dataset and warrant further study to understand how real-time clinical judgment shapes airway selection. Initial device choice may be influenced by patient-level factors, such as suspected aspiration risk, anatomy, or the presence of blood or vomit; clinician-level factors, such as training, comfort, or experience; or system-level elements, such as protocols, equipment availability, or agency culture. For example, some systems may encourage early ETI in patients with contamination risk, while others may promote SGA-first strategies to maximize speed and simplicity. Although aspiration risk is sometimes cited as a reason to favor ETI, both the PART and AIRWAYS-2 trials found no clear difference in overall aspiration rates between airway types.^4,11^ Notably, the AIRWAYS-2 trial^11^ reported higher aspiration after SGA placement but higher aspiration before ETI placement, highlighting that timing and scene dynamics likely influence this relationship. These unknowns also extend to the reasons why clinicians switch, or do not switch, airway devices following failure. Without access to context, such as clinician rationale, scene conditions, or local policy, we cannot assess whether switching behaviors reflect clinical judgment, structural constraints, or habit. These questions would benefit from future mixed-methods research, including prospective studies and qualitative input from EMS clinicians.

A key insight from this analysis is how infrequently airway device switching occurred, even after a first-attempt failure. Only 27 651 ETI-first failures (27.7%) and 3388 SGA-first failures (38.1%) transitioned to a different device. This pattern highlights a strong tendency to continue with the initial airway approach despite unsuccessful placement. While continuing with a particular device may be clinically justified in some situations (eg, continued ETI use with vomit or blood in airway), these findings suggest that switching, particularly switching from ETI to SGA after failure, may be associated with higher overall success. Factors such as protocol structure, clinician familiarity, local practice, experience, training, lack of understanding of SGA safety and effectiveness, or perceived barriers to switching may contribute to the low rate of device changes.^25,26^

The 2020 American Heart Association (AHA) emphasized the importance of tracking overall ETI success rates to guide whether systems should continue using ETI or consider SGA as the initial device for cardiac arrest.^27^ Our findings provide supporting data for this approach in 2 ways: (1) SGA was placed successfully on the first attempt in 178 405 patients (93.0%), and (2) clinicians who switched to SGA following failed ETI had a 94.1% success rate, compared with 70.5% for those who continued with ETI. The AHA also called for regular training and ongoing system-level quality improvement, highlighting a need to address device choice, clinical judgment, and adaptability required to optimize airway management in OHCA settings. These findings reinforce the importance of preparing EMS clinicians in technical skills and in airway decision-making, encouraging reassessment after failed attempts with the goal of optimizing success, which may include switching operators or devices.

Despite evidence supporting the efficacy and ease of use of SGAs, EMS clinicians may continue to prioritize ETI for several reasons.^4,5,6,28^ First, ETI has long been regarded as the gold standard of airway management in both EMS training and culture, often viewed as a marker of clinical competence or advanced skill.^29,30^ This perception may incentivize its use even in situations where more effective alternatives exist. Second, protocols in some jurisdictions may either require or strongly encourage ETI as the initial device, particularly in high-acuity settings like OHCA. Third, some clinicians may believe ETI offers advantages in specific clinical scenarios, such as aspiration risk or airway obstruction, despite limited data demonstrating superiority in OHCA outcomes.^10,28,31^ Additionally, ETI training typically receives more emphasis during initial certification and continuing education, leading to greater familiarity and confidence. These system-level and cultural factors may create inertia against changing airway practices, underscoring the need for ongoing education, protocol reassessment, and data-driven quality improvement to align airway management with evidence-based practices.

In this article, we described airway practice patterns, which included switching devices and multiple airway attempts during OHCA. These occurred in 4.8% of patients in our study. It is important to acknowledge that the goal in resuscitation should remain achieving the highest possible first-pass success through appropriate device selection, preparation, and team coordination, thereby enhancing patient outcome. Since each attempt carries inherent risks, including chest compression interruption, hypoxia, and airway trauma, clinicians should focus on optimizing airway attempts to achieve first-pass success. However, when confronted with the real event of failure, our results underscore that prompt reassessment and adaptation (eg, switching devices or operators) may optimize success, rather than repeated persistence with the same approach. These findings continue to support the ongoing prehospital emphasis on training, preoxygenation, and situational awareness to minimize attempts to ensure effective airway management during OHCA.

### Limitations

This evaluation is subject to several limitations that may impact the accuracy of depicting national practice patterns in advanced airway device order and success. First, reporting bias may occur in documenting procedure attempts and outcomes, as definitions of an attempt (eg, blade insertion vs full device placement) or success (eg, device position vs ventilation confirmation) may vary by clinician and agency. While this heterogeneity introduces variability, the large national sample likely reflects average reporting behavior and mitigates the impact of individual differences in documentation practices, consistent with foundational research on observational study design and prior EMS studies using NEMSIS data.^32,33^

Second, recall bias and incomplete data entry may influence EMS documentation quality. Although 99% of ePCRs are submitted within 10 days of patient contact, delays between care and documentation could affect the accuracy of reported events.^15^ Additionally, missing data, particularly for race and ethnicity (18.8% missing [443 665 of 2 365 224]), limits the ability to examine airway practices across diverse communities.

Third, because outcomes are not available on a national level, this study is procedural in focus and does not assess clinical outcomes, such as return of spontaneous circulation, survival to hospital discharge, or neurological recovery. As such, this analysis does not evaluate how airway device order or switching affects downstream patient outcomes. While procedural success alone cannot substitute for outcome data, understanding airway management trajectories (eg, switching behavior after failed attempts) provides essential insight into clinician decision-making and system performance. These findings identify modifiable areas for quality improvement (eg, low switching rates despite failure) and lay the groundwork for future studies that integrate patient outcomes to assess the clinical implications of these strategies.

Fourth, the rationale for switching or persisting with a particular device is not captured in the dataset. We cannot determine whether decisions to switch airways were driven by patient factors, protocol adherence, clinician training, or scene logistics. This limits the interpretation of observed patterns as either clinically optimal or operationally constrained.

Furthermore, the dataset does not include time-to-placement intervals, CPR quality metrics, or the level of confirmation used for airway success. These unmeasured factors may influence both the likelihood of success and the decision to switch devices.^32^ Additionally, although findings are derived from a large and representative US dataset of 911 EMS activations, they may not generalize to systems outside the United States or specialized response teams with different training models.

## Conclusions

In this cross-sectional study of advanced airway management during OHCA, most patients received an ETI initially, which had a 71.0% success rate. SGA had a 93.0% first-attempt success rate. The initial choice of airway as well as the final choice in the event of first-pass failure appeared to have a major impact on procedural success. The greatest overall success was with a strategy of SGA-first followed by a strategy of switching to SGA in the case of ETI failure. The worst overall strategy was ETI first followed by persistent ETI attempts in the event of an initial failure. These findings highlight that airway management in OHCA should be viewed as a dynamic sequence of decisions. EMS guidelines, education, and quality improvement initiatives should focus on both maximizing first-pass success and clear direction on optimal strategies if the first strategy fails.

## Notes

Supplement.
Data Sharing Statement
