# Supplementary material for: Advanced Airway Device Use Order During Out-of-Hospital Cardiac Arrest
Source: JAMA Netw Open. 2026 Jan 12;9(1):e2553413. doi: 10.1001/jamanetworkopen.2025.53413 (PMC12797093; doi:10.1001/jamanetworkopen.2025.53413)
Supplement: Supplement. — Data Sharing Statement [file jamanetwopen-e2553413-s001.pdf]

Data Sharing Statement

Gage. Advanced Airway Device Use Order During Out-of-Hospital Cardiac Arrest. *JAMA Netw Open*. Published January 12, 2026. doi:10.1001/jamanetworkopen.2025.53413

Data
Data available: Yes
Data types: Deidentified participant data, Data (not involving human participants), Data dictionary
How to access data: All data is available through the publicly available NEMSIS website.
When available: With publication

Supporting Documents
Document types: None

Additional Information
Who can access the data: All data is available through the publicly available NEMSIS website.
Types of analyses: For purposes allowed by the NEMSIS Technical Assistance Center
Mechanisms of data availability: For purposes allowed by the NEMSIS Technical Assistance Center
Any additional restrictions: For purposes allowed by the NEMSIS Technical Assistance Center
